# Effects of Particle Size and Oxide Shell Thickness on the Oxidation Characteristics of Core–Shell Aluminum Nanoparticles Using Molecular Dynamics Simulation

**DOI:** 10.3390/nano16171075

**Published:** 2026-08-29

**Authors:** Siyi He, Zhengqing Zhou, Nan Zhang, Lujia Chai, Qi Liu, Kunpeng Li, Baolin Guo, Lei Ma, Xingci Cheng

**Affiliations:** 1 School of Resources and Safety Engineering, University of Science and Technology Beijing, Beijing 100083, China; 2 State Key Laboratory of Transient Chemical Effects and Control, Xi’an 710061, China; 3 Army Engineering University of PLA, Xuzhou 210001, China

**Keywords:** aluminum nanoparticles, core–shell structure, molecular dynamics simulation, oxidation characteristics

## Abstract

Aluminum nanoparticles (ANPs) possess a core–shell structure, yet the coupled roles of atomic stress and interfacial charge transfer in their slow-heating oxidation remain elusive. This study employs ReaxFF molecular dynamics simulations to investigate the oxidation of six core–shell ANPs with different particle sizes (5–10 nm) and shell thicknesses (0.5–2.0 nm) from 300 K to 1400 K. Results reveal that the stress evolution dictates the oxidation pathway. Thin shells (0.5–1.0 nm) undergo a compressive-to-tensile stress transition, leading to shell rupture at ~1060 K and subsequent outflow and rapid oxidation of Al into clusters, while thick shells (1.5–2.0 nm) maintain compressive confinement, preventing rupture but resulting in incomplete oxidation (58–84%). Mean squared displacement indicates earlier atomic diffusion onset for thin-shell particles (~7 ps) compared to thick-shell ones (~15 ps). Significantly, interfacial charge redistribution provides the electronic driving mechanism: thin shells facilitate charge homogenization and electron loss, lowering diffusion barriers, whereas thick shells sustain distinct charge separation, impeding atomic migration. These findings provide a theoretical basis for the atomic-scale stress–charge–diffusion coupling mechanism, offering crucial insights for the safety assessment and structural design of oxidation-resistant ANPs.

## 1. Introduction

Aluminum powder is an extremely important industrial material characterized by high energy density, low cost, and environmentally benign combustion products, and it is widely employed in catalysts, propellants, explosives, and related fields [1,2,3,4,5,6]. Compared with conventional micron-sized particles, aluminum nanoparticles (ANPs) exhibit lower ignition temperatures, faster burning rates, and enhanced energy release efficiencies [6,7], thereby offering considerable potential for diverse applications. Nevertheless, during preparation, storage, transportation, and processing, ANPs are highly susceptible to forming combustible dust clouds in air, and the heat generated by oxidation may induce spontaneous ignition, consequently triggering severe dust explosion accidents.

The fundamental origin of aluminum dust explosion accidents lies in the pronounced complexity and inherent uncertainty associated with the oxidation behavior of aluminum particles upon heating. Owing to their unique small-size effects and potentially high reactivity, ANPs can undergo oxidation even at ambient temperature, resulting in the rapid formation of an aluminum oxide shell on the particle surface and the establishment of a typical core–shell structure [8,9]. This oxide shell not only diminishes the reactivity of the aluminum particles but may also compromise their energy release efficiency, thereby adversely affecting the performance of propellants and explosives [9,10]. In industrial environments, under heating conditions, the interplay between particle size and initial oxide shell thickness critically influences the oxidation pathways. However, the underlying mechanisms, particularly how these geometric parameters couple with atomic-scale stress and charge redistribution to drive oxidation, remain inadequately understood.

Currently, numerous domestic and international researchers have conducted extensive investigations into the oxidation processes and underlying mechanisms of ANPs through experimental approaches and molecular dynamics simulations. Eisenreich et al. [11,12] investigated the oxidation heating process of ANPs (0.01–25 μm) in air using TGA, SEM, and XRD; the experimental results indicated that, as the oxide shell thickness increases during oxidation, the particle dimensions remain essentially unchanged, with hollow spherical particles being the predominant product. Gao [13] observed the migration of the inner and outer surfaces of the oxide shell on aluminum powder particles at different heating temperatures and found that the shell undergoes marked thickening, with greater inward migration occurring at the inner surface. Zhou et al. [14] employed in situ transmission electron microscopy to reveal the mechanism by which the alumina shell of individual aluminum nanoparticles ruptures at 850 °C due to internal pressure, allowing molten aluminum to escape and rapidly oxidize, thereby forming protrusions. Combined with scanning electron microscopy, they identified particle size as the key factor determining the morphological evolution of the shell. Furthermore, the dynamic shell-rupture and ignition process of individual aluminum nanoparticles under oxidative heating at 10 °C/min was investigated [15], quantitatively revealing that the oxide layer thickness exhibits a three-stage evolution with temperature (initial oxidation, slow oxidation, and vigorous oxidation). Notably, they observed an early precursor of localized significant thickening prior to shell rupture at 770–780 °C. Thomas Cameron et al. [16] reported the synthesis of core–shell Al–SiO_2_ nanoparticles via non-thermal plasma, which exhibited an increased oxidation temperature from 535 °C to 585 °C. For ANPs initially devoid of an oxide shell, Hong et al. [17] employed the ReaxFF reactive force field to study the oxidation and shell formation process of 2.8 nm bare aluminum particles under varying temperatures and oxygen concentrations. Wang [18] investigated the room-temperature oxidation process of bare aluminum particles with diameters of 3, 4, 5, 6, and 7 nm and found that smaller particle sizes correspond to a larger proportion of unsaturated coordination atoms on the surface, leading to more vigorous oxidation reactions. Wang further examined the influence of temperature on the oxide shell [19], elucidated the formation mechanism of chain-like alumina structures at the atomic level, and observed the outward migration of internal Al atoms resulting in the formation of internal cavities. Zeng et al. [20] conducted molecular dynamics simulations to study the atomic diffusion behavior of core–shell structured ANPs during heating and proposed that the inward diffusion of oxygen atoms within the shell constitutes the primary cause of the initial reaction at the core–shell interface. Li et al. [21] constructed models of ANPs with core diameters ranging from 20 to 40 nm and a fixed alumina shell thickness of 3 nm, and systematically investigated the influence of particle size on the oxidation behavior. 

In summary, most existing experimental studies have focused on bare aluminum particles or simply described the shell breakage phenomenon with particle sizes/shell thickness rather than considering the simultaneous interplay between oxide shell thickness and atomic-scale stress/charge redistribution. However, studies on a systematic elucidation of the coupling mechanism between atomic-scale stress evolution, interfacial charge transfer, and atomic diffusion remain comparatively limited. In this work, ReaxFF-based MD simulations was employed to systematically investigate the decoupling of stress evolution and charge redistribution, proving that interfacial charge redistribution is not merely a passive consequence of oxidation, but rather an active electronic driving force. In particular, charge homogenization across the core–shell interface reduces the electrostatic potential barrier for Al detachment, and the degree of this homogenization correlates with the onset time of atomic diffusion, as reflected by mean squared displacement (MSD). The charge redistribution is not merely a phenomenon; it is a mechanistic advance because it explains why the diffusion barrier decreases (lowering electrostatic barrier) in thin-shell particles, which fundamentally drives the diffusion phenomena. Our findings offer a theoretical basis for understanding the safety risks of ANPs and can guide the structural design of oxidation-resistant aluminum nanomaterials.

## 2. Model Establishment and Parameter Setting

### 2.1. Model Establishment

**Figure 1:**
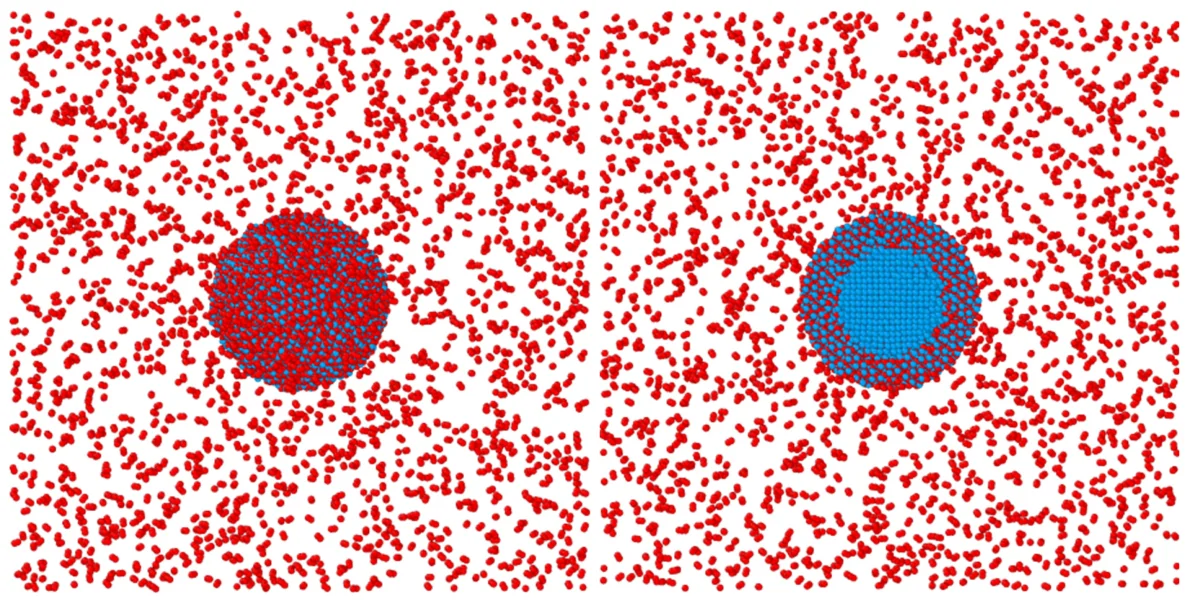
C2S1 model diagram.

In view of the fact that nano-aluminum particles exhibit a core–shell structure at room temperature, the oxide layer thickness was set with reference to the work of Sundaram, according to which the oxide layer thickness for aluminum particles with diameters of 5–10 nm ranges from 0.5 to 2 nm [22]. Accordingly, aluminum particles with diameters of 6, 8, and 10 nm were constructed, and their oxide layer thicknesses were set to 1, 1.5, and 2 nm, respectively. To facilitate model identification, each configuration is designated by the core radius C (Core) and the shell thickness S (Shell). For instance, an aluminum particle with a diameter of 6 nm, a shell thickness of 1 nm, and an aluminum core radius of 2 nm is denoted as C2S1. Aluminum atoms are colored blue and oxygen atoms red; a representative model is illustrated in Figure 1.

However, for aluminum particles with diameters smaller than 10 nm, the oxide layer thickness is not a fixed value but rather falls within a range, and current research has yet to determine the oxide layer thickness for each specific size of nano-aluminum particles. Therefore, to more comprehensively simulate the oxidation behavior of these particles, the aforementioned three models were extended: while keeping the aluminum core diameter unchanged, the oxide layer thickness in each model was halved, yielding a group of models with thinner oxide shells. Consequently, two sets of models—thin-shell and thick-shell—were obtained, and the detailed parameters are presented in Table 1.

**Figure 2:**
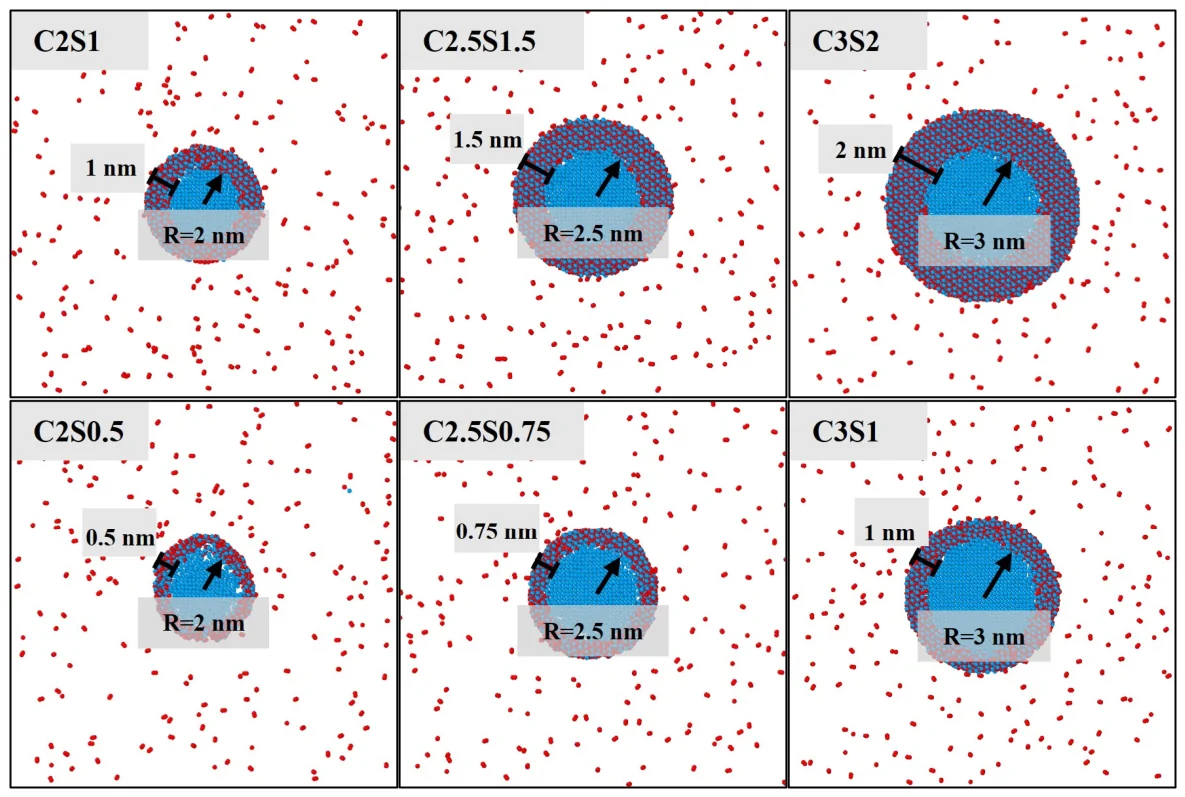
Section diagram of six aluminum particle models.

Based on the unit cells of amorphous Al_2_O_3_ and face-centered cubic (FCC) Al, supercell expansion was performed using the ATOMSK (v 0.12) molecular modeling software. Pure aluminum core models and hollow alumina shell models with corresponding radii were constructed separately according to the specified aluminum core radii and oxide shell thicknesses. Subsequently, the aluminum cores were placed into the hollow alumina shells, resulting in six constructed models, as illustrated in Figure 2.

Since the aluminum core and the oxide shell were modeled separately and then placed together, a gap existed between them, and the core and the shell initially formed two distinct entities. To promote better integration of the core–shell structure, to more closely approximate the actual configuration of nano-aluminum particles, and to enhance the stability of the model system, the constructed particle model was relaxed at 300 K for 300 ps, yielding a structurally stable nano-aluminum particle with an alumina shell.

### 2.2. Parameter Setting

Traditional empirical force fields require the predefinition of bonding relationships between atoms prior to molecular dynamics simulations, which precludes the modeling of bond breaking and formation during chemical reactions. The ReaxFF force field was first introduced by Van Duin et al. [23], with parameters derived from experimental data and quantum mechanical principles to investigate complex reactions in hydrocarbons. It is constructed on the basis of bond order, thereby circumventing explicit bonds and allowing for continuous bond formation and dissociation. At present, ReaxFF has been developed for a broad range of reactive chemical systems.

In MD simulations, due to the limitations of the computing power of the workstation, the time step is usually set to femtoseconds. The entire simulation process takes only a few nanoseconds to complete, which is far below the time scale of real life. If the simulation is conducted according to the ignition rate in reality, it would be extremely difficult. The required computing resources, time, and the accuracy of the calculation results cannot be guaranteed. Yang et al. [24] demonstrated through parametric analysis that heating rates ranging from 0.01 to 0.1 K/fs (i.e., 10–100 K/ps) are sufficient to maintain the system in a quasi-equilibrium state. Accordingly, to simulate the oxidation of ANPs at a relatively low heating rate while ensuring computational feasibility, a uniform heating rate of 20 K/ps was adopted for all simulations in this study. With respect to the integration time step, preliminary tests indicated that a time step of 0.2 fs is adequate to accurately capture the adsorption, dissociation, and diffusion processes of oxygen molecules during heating to 1400 K. This value has been further corroborated as appropriate by multiple previous studies [17,25,26].

All molecular dynamics simulations of aluminum nanoparticle oxidation were performed using the LAMMPS package (3 Mar 2020), and visualization and post-processing analyses were conducted employing the OVITO software (v 3.14.1). The simulation domain was configured as a cubic box with dimensions of 200 Å × 200 Å × 200 Å, subject to periodic boundary conditions in all three spatial directions. The box contained 4000 oxygen molecules, and the pre-constructed aluminum nanoparticle model was positioned at the center of the simulation box. The heating process was carried out within the microcanonical ensemble (NVE). The equations of motion were integrated using the Velocity-Verlet algorithm to ensure conservation of total energy. Temperature regulation was achieved via a Berendsen thermostat, with a temperature damping parameter set to 100 fs.

It is important to note that the atomic charges calculated by ReaxFF are not absolute physical quantities but are parameterization-dependent. Therefore, in the following discussions, we focus on relative charge trends and systematic comparisons between thin-shell and thick-shell models under identical force-field parameters and simulation conditions, rather than on absolute charge values. This conservative approach ensures that our conclusions about charge-related effects are drawn from consistent and reproducible contrasts.

### 2.3. Results Determination and Post-Processing Methods

To ensure the reproducibility of this study and to explicitly clarify how the analytical results were determined, a detailed description of the primary data generation and post-processing procedures using LAMMPS and OVITO is provided: 

(1) Primary data generation using LAMMPS:

All ReaxFF molecular dynamics simulations in this study were performed using the LAMMPS software. LAMMPS is responsible for generating the primary simulation trajectories and raw data. Specifically, the atomic stress tensors are computed and recorded at each step based on the virial theorem. The mean squared displacement (MSD) of atoms is directly obtained using the built-in compute msd command in LAMMPS. The dynamic partial charges of the atoms are determined at each time step through the charge equilibration method integrated into the ReaxFF force field.

(2) Post-processing and data extraction using OVITO:

All simulation trajectories (dump files) output by LAMMPS were subsequently analyzed and visualized using the OVITO software. The specific data extraction procedures for each result are as follows:

Morphological evolution and shell rupture analysis: The slice function within OVITO was utilized to generate 1-nm-thick cross-sectional views centered at the particle center. This allowed for the intuitive observation of internal void formation, morphological changes, and shell breakage.

Extraction of stress evolution: Using the compute stress/atom command in LAMMPS, atomic stress tensors are extracted based on the virial theorem. Subsequently, custom Python (v 3.7.0)scripts are used to precisely divide atoms into the Al core and Al_2_O_3_ shell regions, and the atomic stress tensors in these two regions are averaged separately to obtain the stress evolution curves at different temperatures.

Atomic diffusion (MSD) analysis: Based on the MSD data output by LAMMPS, separate statistical analyses were performed for different atom types (i.e., Al atoms in the core and O atoms in the shell) using OVITO. The onset time and diffusion rates of atomic migration were determined by plotting the MSD curves.

Pair distribution function (PDF) calculations: The built-in coordination analysis module in OVITO was used to calculate the radial distribution functions (RDFs) for Al-Al and Al-O pairs. Different cutoff radii were selected at various temperature steps for quantitative analysis of lattice amorphization and bond length variation.

Atomic charge distribution mapping: OVITO was used to render the atoms based on their calculated charge values with a color-mapping scheme. Since ReaxFF dynamically updates the atomic charge states, different charge states were assigned colors: positively charged ions were colored red, negatively charged ions were colored blue, and neutral atoms were colored green.

By systematically coupling primary data generated from LAMMPS with scientific post-processing and extraction via OVITO, it was ensured that all conclusions in this study were derived from a unified, well-defined, and reproducible analytical framework.

## 3. Results and Discussion

### 3.1. Effect of Oxide Shell Thickness on the Morphological Evolution of 5–10 nm Aluminum Particles

**Figure 3:**
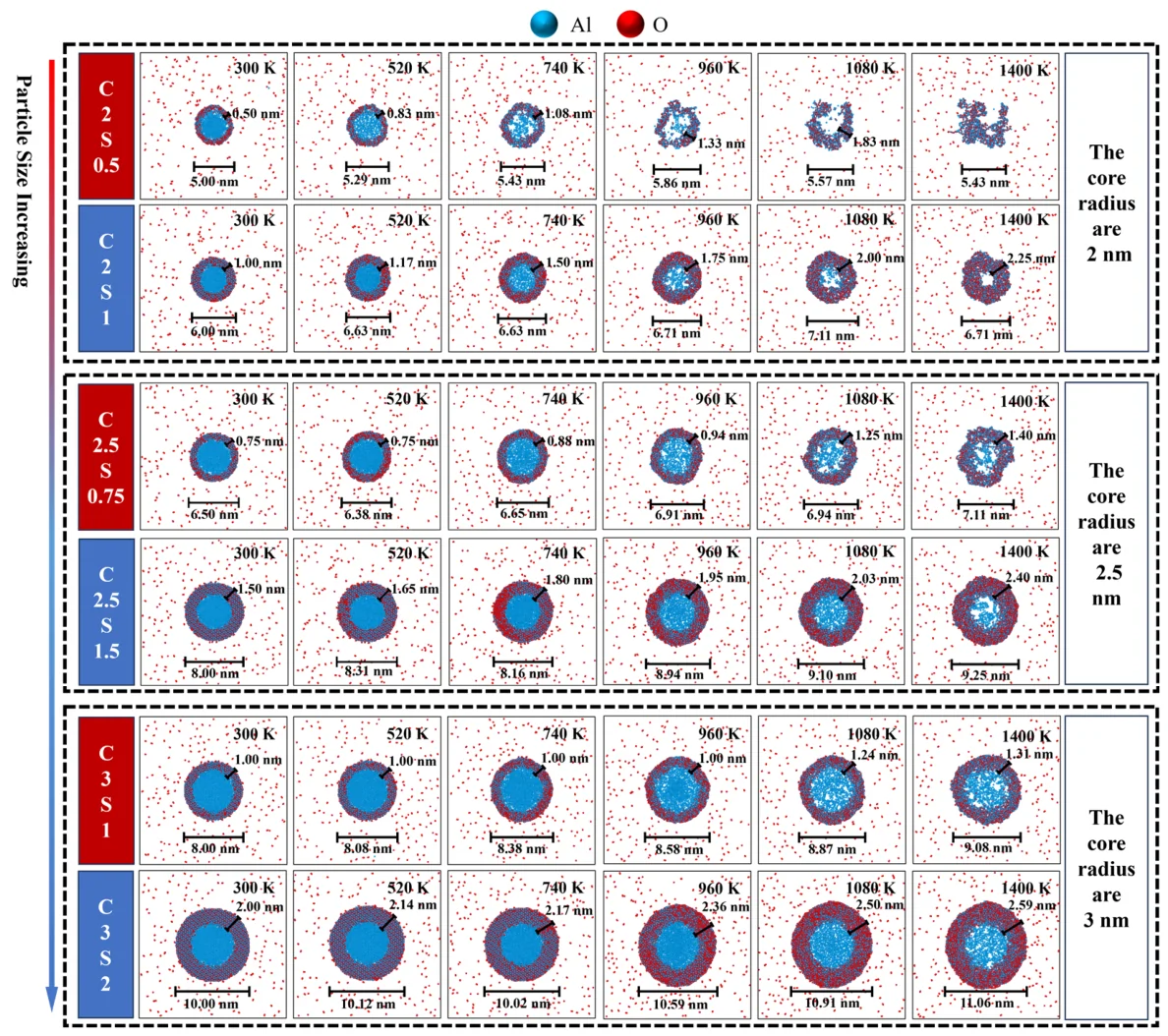
Morphological changes in six aluminum particle models.

To clearly illustrate the dynamic internal morphological changes in the six models during heating from 300 K to 1400 K at a rate of 20 K/ps, the entire oxidation process was divided into five equal stages. At temperatures of 300 K, 520 K, 740 K, 960 K, 1180 K, and 1400 K, 1-nm-thick slices were taken with the particle center as the origin, allowing clear visualization of the internal atomic evolution. The morphological changes were likewise classified into a thick-shell group (blue) and a thin-shell group (red). As shown in Figure 3, the six models illustrate the oxidation processes of aluminum particles with different diameters and oxide shell thicknesses.

At the initial state, these aluminum particles exhibit a close-packed atomic arrangement; the aluminum core possesses a regular face-centered cubic (FCC) structure, while the outer layer displays an amorphous alumina structure. For C2S1, as the temperature increases, the Al atoms in the aluminum core decrease significantly, and the particle is completely oxidized at 1400 K, forming an internal cavity without any shell rupture, which indicates that Al atoms diffuse outward during heating. The C2.5S1.5 particle undergoes slight deformation upon heating, and a portion of the aluminum inside remains unoxidized, whereas the C3S2 particle shows no obvious deformation and retains more unoxidized aluminum. A comparison of the three models reveals that under the same conditions, smaller particles are more easily oxidized, and larger particles may require higher temperatures or longer times to achieve complete oxidation. Compared with the thick-shell group, the thin-shell group exhibits more pronounced morphological changes. For the C2S0.5 particle, the alumina layer becomes discontinuous at 1180 K, indicating that the oxide shell has ruptured, and by 1400 K it transforms into agglomerated alumina. The C2.5S0.75 particle is not fully oxidized during the process; at 1400 K, its morphology becomes irregularly spherical, with a non-uniform oxide shell thickness and a roughened surface. Similarly, the C3S1 particle is not fully oxidized, undergoes slightly less deformation, but also becomes irregularly spherical at 1400 K. Comparing the left and right panels of Figure 3 reveals the effect of oxide shell thickness on the oxidation phenomena of nano-aluminum particles. These oxidation phenomena can be classified into three types: particles with ruptured oxide shells, hollow particles, and slightly deformed particles. Both the C2S1 and C2S0.5 particles are completely oxidized; the former becomes a hollow particle, whereas the oxide shell of the latter ruptures. The remaining four particles are not fully oxidized, but those with thinner oxide shells exhibit markedly higher degrees of oxidation and particle deformation than those with thicker shells. Therefore, a thicker oxide shell makes the oxidation reaction more difficult to proceed.

**Figure 4:**
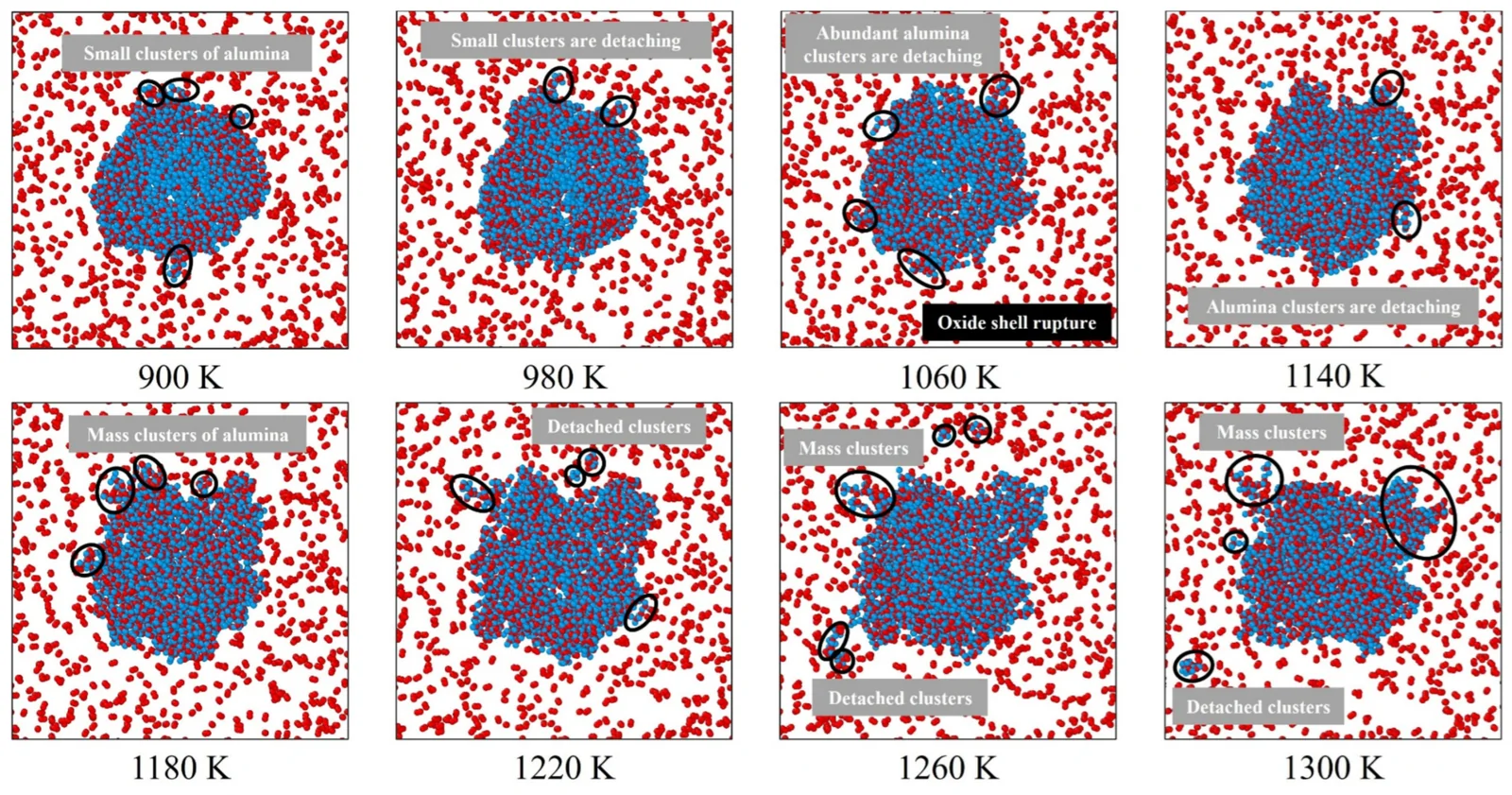
The overall morphological changes in C2S0.5 at high temperature.

To observe the rupture of the alumina shell in the C2S0.5 particle, the high-temperature oxidation process of this model was carefully examined, as shown in Figure 4. At 900–1140 K, small alumina clusters appear on the particle surface and grow progressively. This phenomenon resembles the chain-like alumina structures observed by Zhang et al. [27] on bare aluminum particles, which originate from the preferential oxidation of low-coordinated Al atoms on the particle surface. However, in the present model, an alumina layer already exists on the particle surface, and starting from 1060 K, alumina clusters continuously detach from the surface, indicating that these surface clusters are not chain-like alumina. Before the oxide shell ruptures, it experiences tensile stress and gradually develops cracks, through which molten aluminum flows out and is subsequently oxidized. In the simulation of the C2S0.5 particle, the alumina clusters appearing on the surface before 1060 K are formed by aluminum permeating outward from the interior and being oxidized by oxygen. After 1060 K, the clusters detaching from the particle surface likely result from oxide shell rupture, which allows aluminum to diffuse outward and undergo rapid oxidation. As the temperature rises, more aluminum diffuses out of the particle, leading to a greater amount of oxidized alumina clusters.

### 3.2. Effect of Oxide Shell Thickness on the Diffusion Behavior of 5–10 nm Aluminum Particles

#### 3.2.1. Effect of Oxide Shell Thickness on the Number of Aluminum Atoms

**Figure 5:**
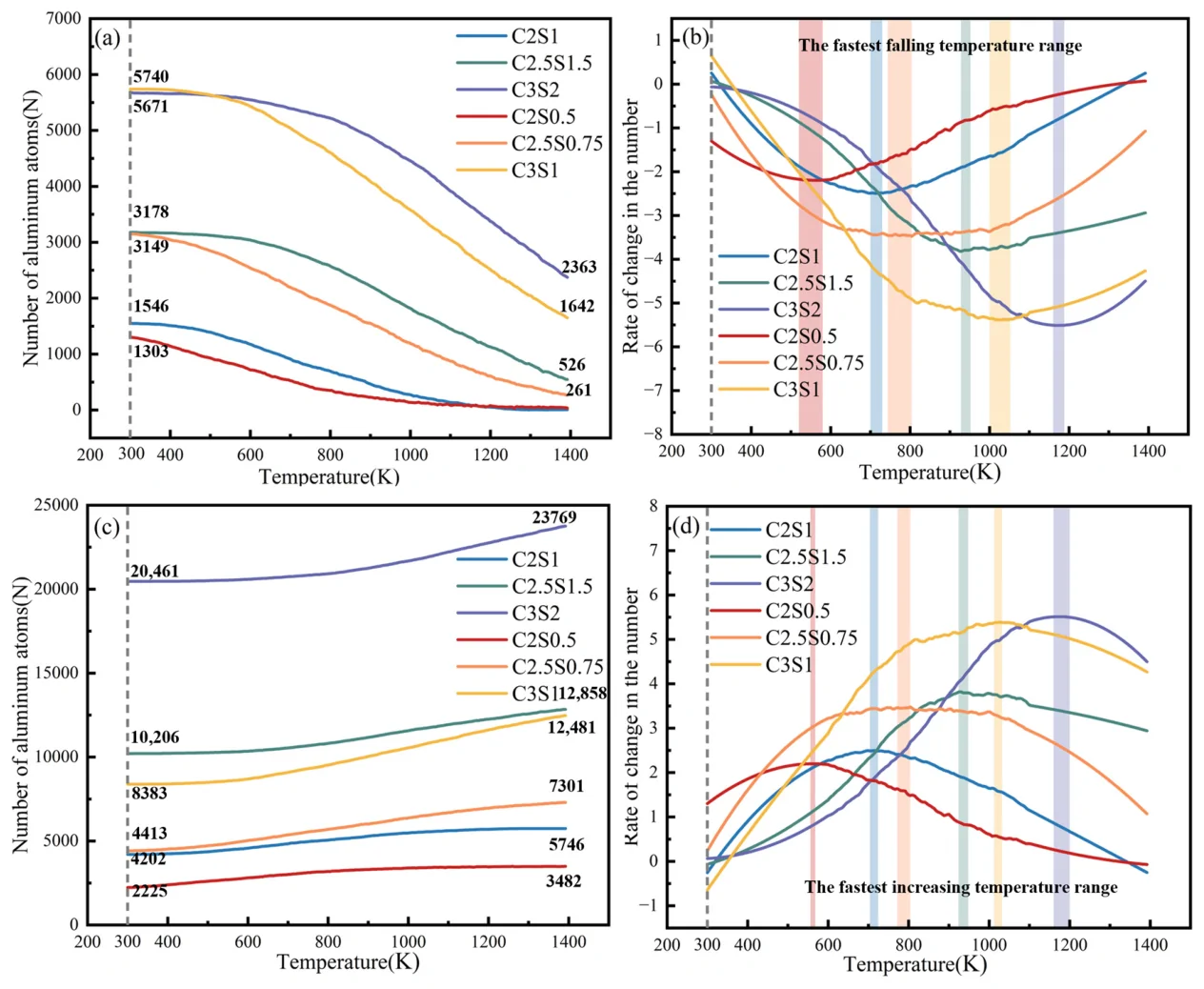
The number and rate of change in Al atoms in aluminum core (top row) and oxide shell. (**a**) the number of Al atoms in aluminum core; (**b**) the rate of change in Al atoms in aluminum core; (**c**) the number of Al atoms in oxide shell; (**d**) the rate of change in Al atoms in oxide shell;

To investigate the diffusion behavior of atoms within the particle during oxidation, aluminum atoms were classified into two categories: those in the aluminum core and those in the oxide shell. Whether an individual Al atom formed a chemical bond with an oxygen atom served as the criterion for distinguishing the core from the shell. The temporal evolution of the number of Al atoms in the aluminum core and in the oxide shell is presented in Figure 5, where cool-colored curves correspond to the thick-shell group and warm-colored curves to the thin-shell group.

As shown in Figure 5a, the number of Al atoms in the aluminum core exhibits a decreasing trend during the oxidation process for all six models. A smaller core radius is associated with a smaller decline in magnitude and consequently fewer Al atoms consumed. For the C2S1 and C2S0.5 particles, which possess the smallest core radii, the number of Al atoms within the core approaches zero in the temperature range of 1200 K to 1400 K, and the oxidation fraction of Al atoms is essentially 100%. In contrast, the oxidation fractions for the C2.5S1.5, C2.5S0.75, C3S2, and C3S1 particles are 84%, 92%, 58%, and 72%, respectively. These results indicate that under identical heating conditions, larger aluminum particles exhibit a lower oxidation fraction of Al atoms and are therefore more resistant to oxidation. For particles with identical core sizes, a thicker oxide shell impedes the oxidation process more significantly; i.e., a thicker oxide shell retards the oxidation of the nano-aluminum particle Regarding the rate of change in the number of Al atoms in the aluminum core (see Figure 5b), all six models display an initial decrease followed by an increase. However, since the total number of Al atoms is decreasing, the rate of change remains negative throughout. A comparison of the temperature ranges at which the number of Al atoms declines most rapidly reveals that larger particles and thicker oxide shells correspond to slower oxidation of the aluminum core, consistent with the morphological evolution of the particles described earlier.

In contrast to the trend observed in Figure 5a, Figure 5c shows that the number of aluminum atoms in the oxide layer increases during oxidation for all six models, confirming that Al atoms from the core migrate into the oxide shell upon heating. Specifically, the number of Al atoms in the oxide layer of the C2S1, C2S0.5, C2.5S1.5, C2.5S0.75, C3S2, and C3S1 particles increases by 37%, 56%, 26%, 66%, 16%, and 49%, respectively. These findings demonstrate that under identical heating conditions, larger aluminum particles exhibit a lower proportion of Al atoms diffusing into the oxide shell. Likewise, for particles with equivalent core sizes, a thicker oxide shell results in a lower proportion of Al atoms migrating into the shell, further indicating a greater resistance to oxidation. This conclusion aligns with the analysis of atomic diffusion within the aluminum core. The rate of change in the number of Al atoms in the oxide shell (see Figure 5d) is the exact opposite of that in the aluminum core, further substantiating that Al atoms from the core diffuse into the shell during heating and become oxidized, thereby contributing to shell growth.

#### 3.2.2. Effect of Oxide Shell Thickness on Active Aluminum Content

**Figure 6:**
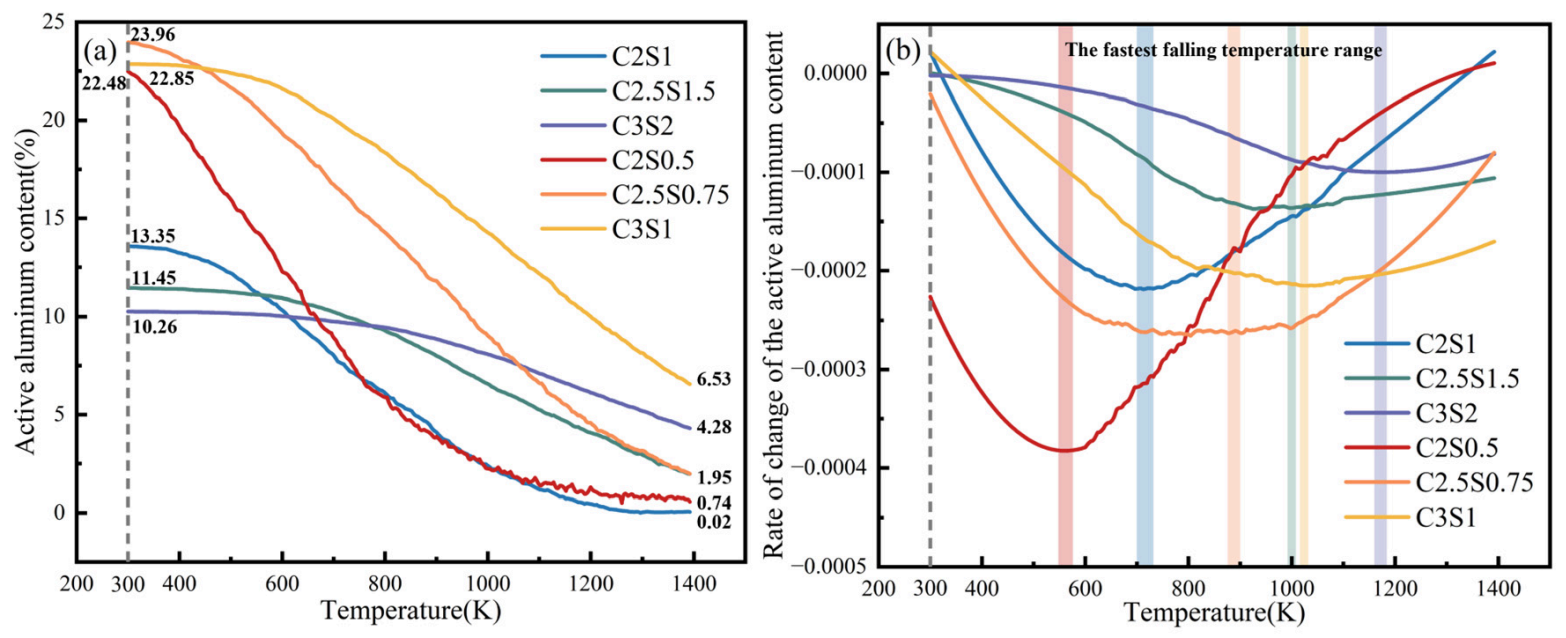
The activity aluminum content and rate of change in six aluminum particle models in oxidation. (**a**) the activity content and rate of change in six aluminum particle models in oxidation; (**b**) the rate of change of the active aluminum content in six aluminum particle models in oxidation;

The active aluminum content at different times and temperatures was calculated based on the number of aluminum and oxygen atoms during the oxidation process in the six models, as shown in Figure 6. Particles with a thicker oxide shell exhibit lower active aluminum content, whereas those with a thinner oxide shell retain higher active aluminum content. Morphological evolution of the C2S1 and C2S0.5 particles reveals that they are nearly completely oxidized at 1400 K. Consistently, Figure 6a shows that the active aluminum content of C2S1 and C2S0.5 particles reaches zero, confirming the agreement between the observed phenomenon and the quantitative data. As illustrated in Figure 6b, the three models in the thin-shell group exhibit a more pronounced and rapid decline in active aluminum content. Moreover, in both groups, the decrease in active aluminum content is accelerated as the particle size becomes smaller. These findings indicate that a thinner oxide shell and a smaller particle size both contribute to an enhanced oxidation rate.

#### 3.2.3. Effect of Oxide Shell Thickness on the Diffusion of Different Atoms

**Figure 7:**
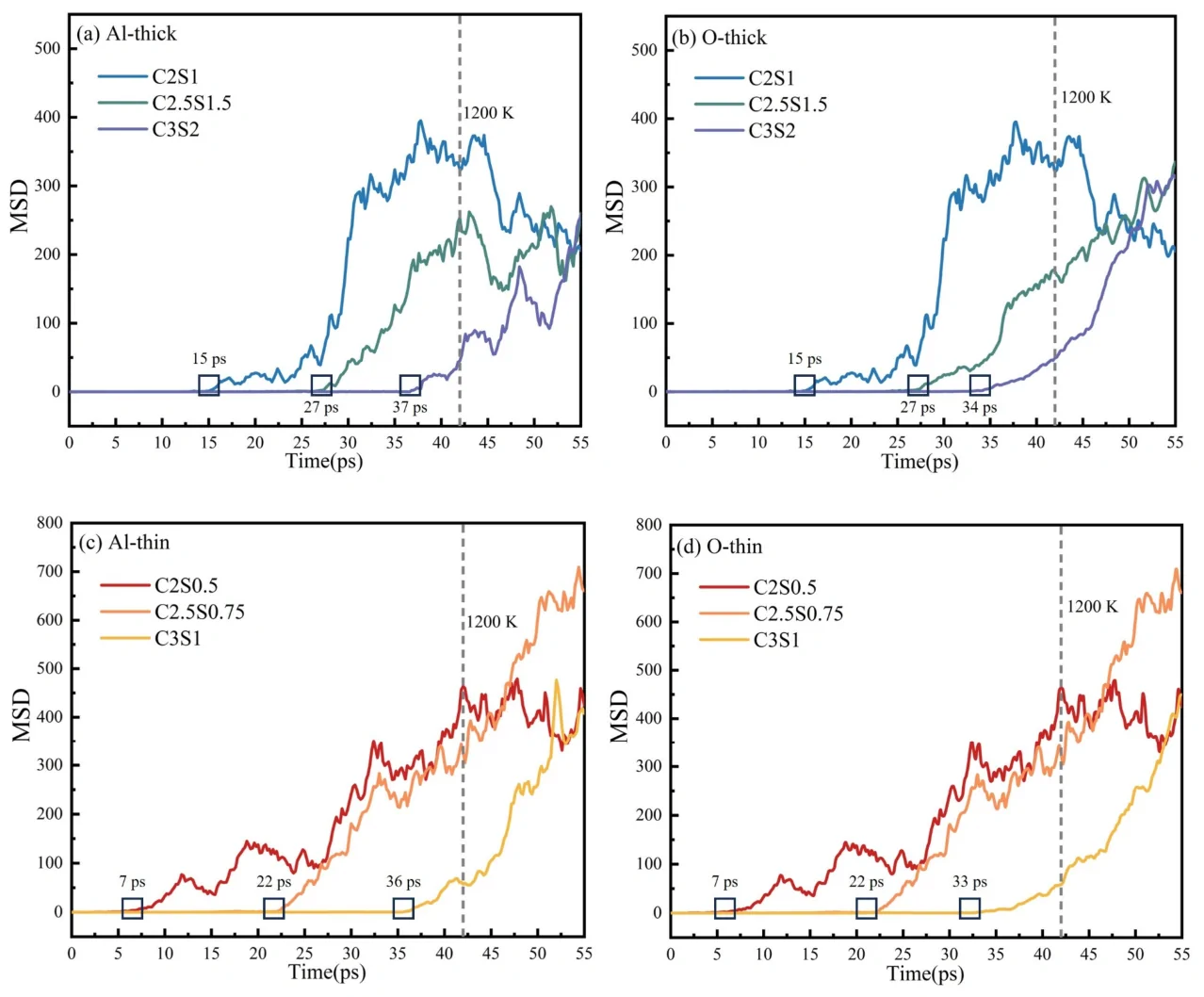
MSD of aluminum in the core and oxygen in the shell of six aluminum particle models.

The mean squared displacement (MSD) is defined as a measure of the deviation in the position of a molecule or atom in a liquid or solid relative to a reference position over a given period of time. During the oxidation of nano-aluminum particles, the MSD scatter plots for aluminum atoms in the core and oxygen atoms in the shell characterize their respective diffusion and migration behaviors throughout the oxidation process, as illustrated in Figure 7.

As can be observed from Figure 7a,c, the aluminum atoms within the core remain relatively stable during the initial heating stage. With increasing temperature, the MSD values rise progressively. In the thick-shell group, the diffusion of aluminum atoms commences as early as 15 ps, whereas in the thin-shell group, the onset occurs even earlier, at approximately 7 ps. Both groups exhibit a consistent trend wherein a smaller particle size correlates with an earlier initiation of Al atom diffusion. For particles possessing identical aluminum core dimensions, a thinner oxide shell leads to earlier diffusion of Al atoms from the core, suggesting that the oxide shell impedes heat conduction to a certain extent: a thicker shell prolongs the time required for heat transfer to reach the core, resulting in a slower temperature rise in the core and a delayed atomic vibration response, thereby causing the MSD curve fluctuations to appear at later times. Furthermore, owing to their relatively small particle sizes, both the C2S1 model in the thick-shell group and the C2S0.5 model in the thin-shell group are essentially completely oxidized at 1200 K (corresponding to 42 ps), after which their MSD values cease to increase. A comparison between the C2S1.5 and C2S0.75 particles reveals that the former exhibits fluctuations without further increase beyond 1200 K, whereas the latter continues to rise, indicating that a thicker oxide shell also hinders the diffusion of Al atoms within the core.

A comparative examination of the four panels in Figure 7 reveals that the diffusion behavior of oxygen atoms within the oxide shell closely parallels that of the aluminum atoms, underscoring that both aluminum and oxygen diffusion play equally significant roles during the heating of nano-aluminum particles. In the C2S1 and C2S0.5 models, the aluminum particles are nearly fully oxidized at 1200 K, leaving no excess Al atoms available for further reaction; consequently, oxygen atoms are confined to diffusion solely within the oxide layer. In contrast, for the C2.5S1.5, C3S2, and C3S1 particles, the MSD of O atoms displays a continuously increasing trend without discernible fluctuations. This phenomenon arises because the outward diffusion of Al atoms is retarded by the oxide shell, whereas O atoms, by virtue of their smaller size, diffuse more readily. In the thin-shell group, the relatively thin oxide shell permits rapid diffusion of oxygen atoms into the interior of the aluminum particle, leading to a swift oxidation reaction and a comparatively steep initial rise in the MSD curve. Conversely, in the thick-shell group, the thicker oxide shell imposes a longer diffusion pathway and a slower diffusion rate for oxygen atoms, thereby rendering the oxidation process more stable.

Notably, the earlier onset of Al diffusion in thin-shell particles (~7 ps) correlates with the rapid charge homogenization observed in Section 3.5 (occurring at ~960 K), whereas thick-shell particles exhibit both delayed diffusion (~15 ps) and persistent charge separation even at 1400 K. This consistent temporal correspondence across independent observables supports the view that interfacial charge redistribution lowers the electrostatic barrier for atomic transport.

### 3.3. Analysis of Stress Evolution in 5–10 nm Aluminum Particles with Different Oxide Shell Thicknesses

**Figure 8:**
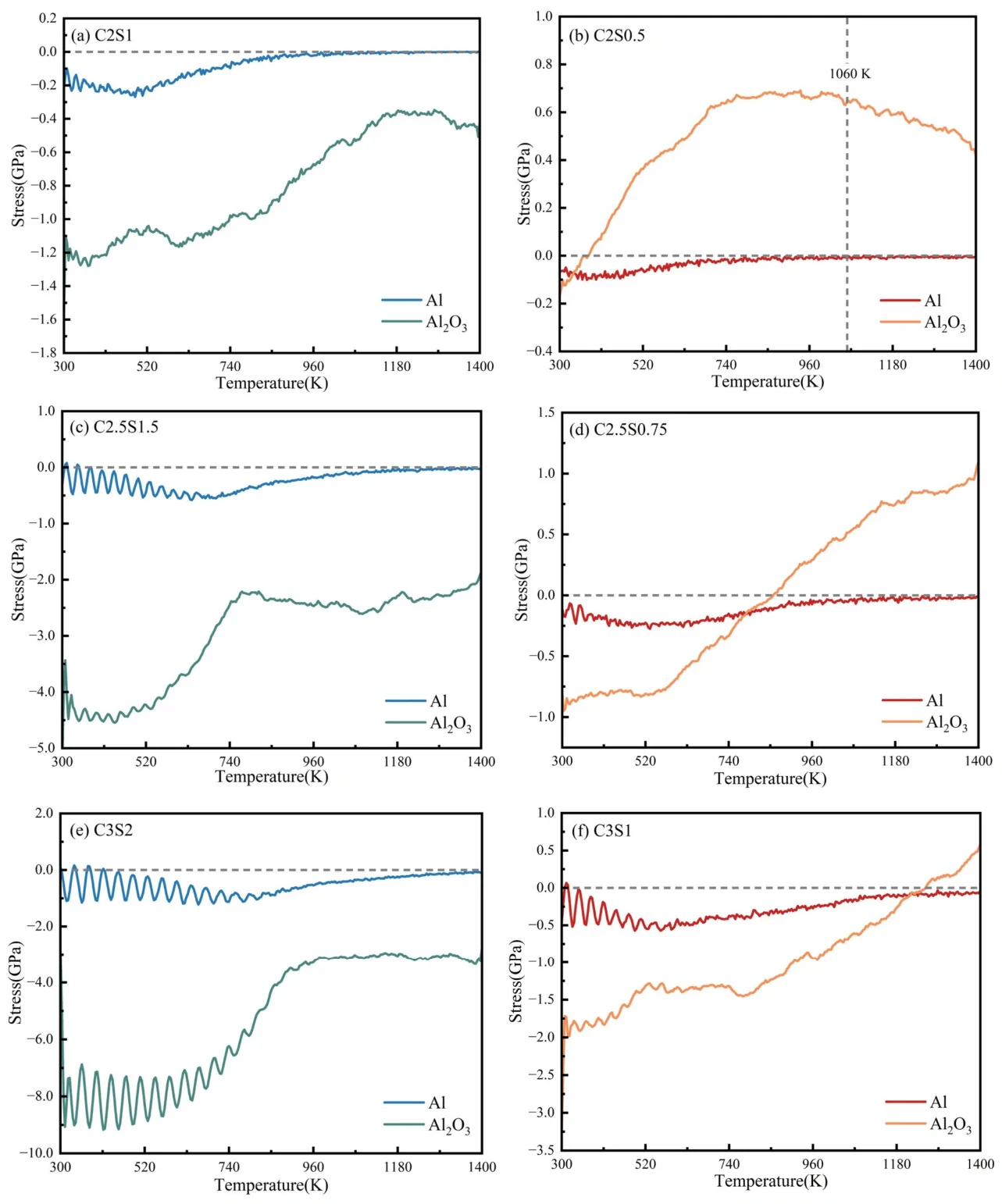
Stress–temperature curves of the Al core and the Al_2_O_3_ shell during the oxidation of (**a**) C2S1, (**b**) C2S0.5, (**c**) C2.5S1.5, (**d**) C2.5S0.75, (**e**) C3S2 and (**f**) C3S1.

The internal stress within the core–shell structure serves as a critical driving force for morphological evolution and atomic diffusion. Figure 8a–f depict the evolution of average stress within the aluminum core and the oxide shell during the heating process.

For particles with thin oxide shells (C2S0.5, C2.5S0.75, and C3S1), the initial compressive stress on the alumina shell rapidly transitions into tensile stress as temperature increases (e.g., above ~900 K in Figure 8d for C2.5S0.75). This tensile stress is primarily induced by two factors: the outward diffusion of molten Al atoms, which exerts internal pressure on the shell, and the thermal expansion mismatch between the Al core and the Al_2_O_3_ shell. When the localized tensile stress exceeds the mechanical strength of the thin amorphous alumina, the shell ruptures (observed at ~1060 K for C2S0.5). Once rupture occurs, the outward flow of Al becomes unrestricted, leading to immediate exposure to ambient oxygen and forming detached alumina clusters. Concurrently, the stress in the aluminum core of these thin-shell particles decreases monotonically as the core depletes and the confinement weakens, although it does not fully vanish due to residual aluminum.

Conversely, for particles with thick shells (C2S1, C2.5S1.5, C3S2), the external alumina shell exerts strong mechanical confinement. The compressive stress on the shell gradually decreases with temperature due to thermal relaxation but tends to remain compressive, as shown in Figure 8. The thick shell maintains structural integrity throughout the process. This continuous compressive constraint suppresses the internal pressure buildup, preventing explosive rupture. However, this mechanical confinement simultaneously impedes the outward diffusion of aluminum atoms and inward diffusion of oxygen, resulting in a much slower oxidation rate. The sustained compressive stress explains why thick-shell particles undergo incomplete oxidation and maintain an internal Al core at 1400 K.

In summary, the competition between the outward diffusion pressure of the core and the confining stress of the shell dictates the oxidation pattern. A thinner shell favors a tensile-to-rupture pathway, while a thicker shell sustains compressive constraint, thereby hindering further atomic diffusion and reaction.

### 3.4. Analysis of Pair Distribution Function (PDF) in 5–10 nm Aluminum Particles with Different Oxide Shell Thicknesses

**Figure 9:**
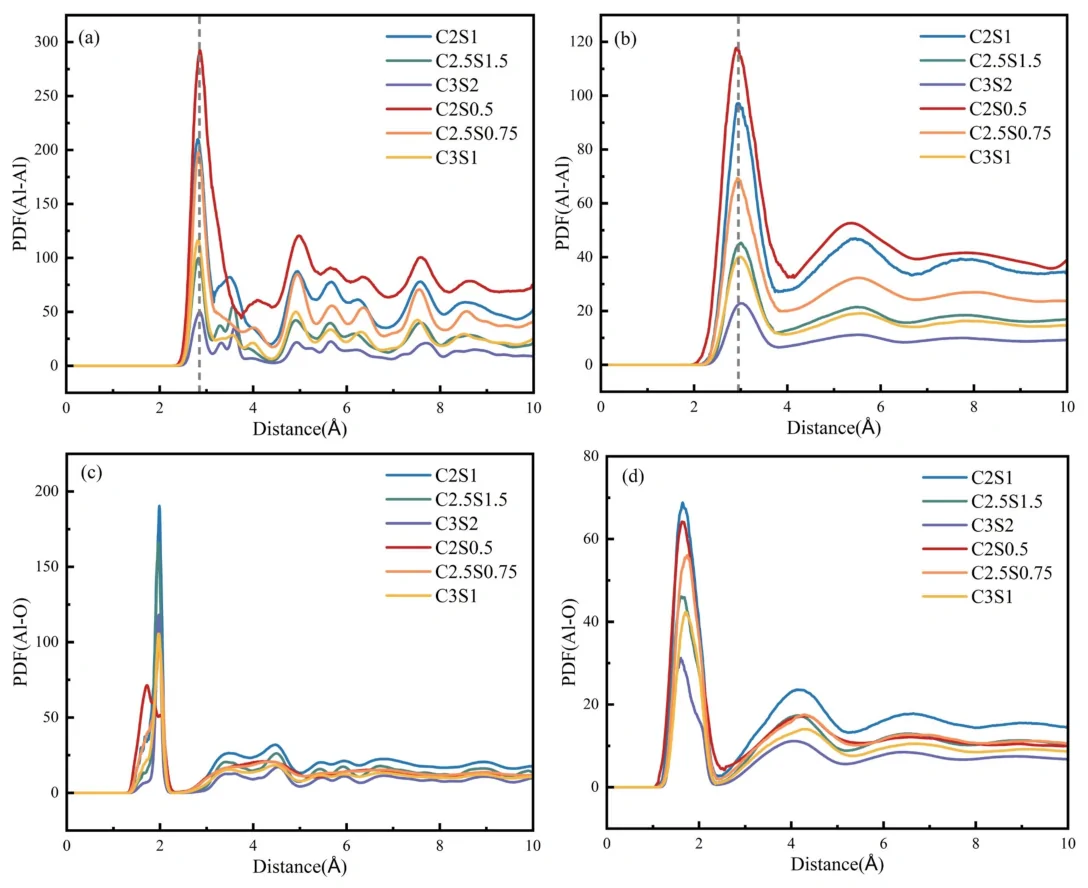
Al–Al and Al–O PDF of the initial ANPs (left column) and their final products (right column). (**a**) Al–Al PDF of the initial ANPs; (**b**) Al–Al PDF of their final products; (**c**) Al–O PDF of the initial ANPs; (**d**) Al–O PDF of their final products;

In the pair distribution function (PDF) plots, the cool-toned (blue) curves correspond to the thick-shell group, whereas the warm-toned (red) curves represent the thin-shell group. In the initial state, the Al–Al PDF profiles of all six particles exhibit a sharp, high-intensity peak at approximately 2.85 Å (Figure 9a), which is consistent with the Al–Al bond length in face-centered cubic (FCC) aluminum. In the final oxidation products, the PDF peaks become lower in intensity and broader in width (Figure 9b), indicating the transformation of crystalline Al into amorphous Al_2_O_3_ accompanied by an increase in structural disorder. Concurrently, the Al–Al bond length increases to 2.95 Å, which can be attributed to the outward migration and oxidation of aluminum atoms toward the shell region as well as thermally induced lattice expansion and distortion [18]. Compared with the initial state, the intensity of the first-neighbor peak decreases markedly for all particles; the relative reductions observed for the thin-shell group (59.93%, 64.97%, and 65.22%, respectively) are consistently larger than those for the thick-shell group (53.59%, 54.55%, and 56%, respectively). This discrepancy arises because the faster inward diffusion of oxygen atoms in the thin-shell group promotes a more extensive conversion of crystalline aluminum into amorphous alumina, rendering the peak attenuation a more direct indicator of the true oxidation extent. Although a larger aluminum nanoparticle size generally results in a thicker oxide shell and a greater reduction in the first-peak intensity, this more pronounced intensity loss is primarily driven by severe lattice distortion and amorphization within the Al core induced by the substantial internal stress imposed by the confining oxide shell, rather than signifying a more complete oxidation process. The positions of the highest peaks in Figure 9c indicate that the initial Al–O bond lengths for the various ANPs fall in the range of 1.88–1.92 Å. In the final oxidation products, these bond lengths shorten to approximately 1.8 Å (Figure 9d), a value that is in agreement with that reported for amorphous alumina [28], thereby suggesting that elevated temperatures enhance the short-range order of the oxide shell and strengthen the Al–O bonds, leading to a more stable and compact local structure. Meanwhile, the decreased peak intensities and broadened peak widths reflect an overall increase in disorder caused by enhanced thermal atomic vibrations.

**Figure 10:**
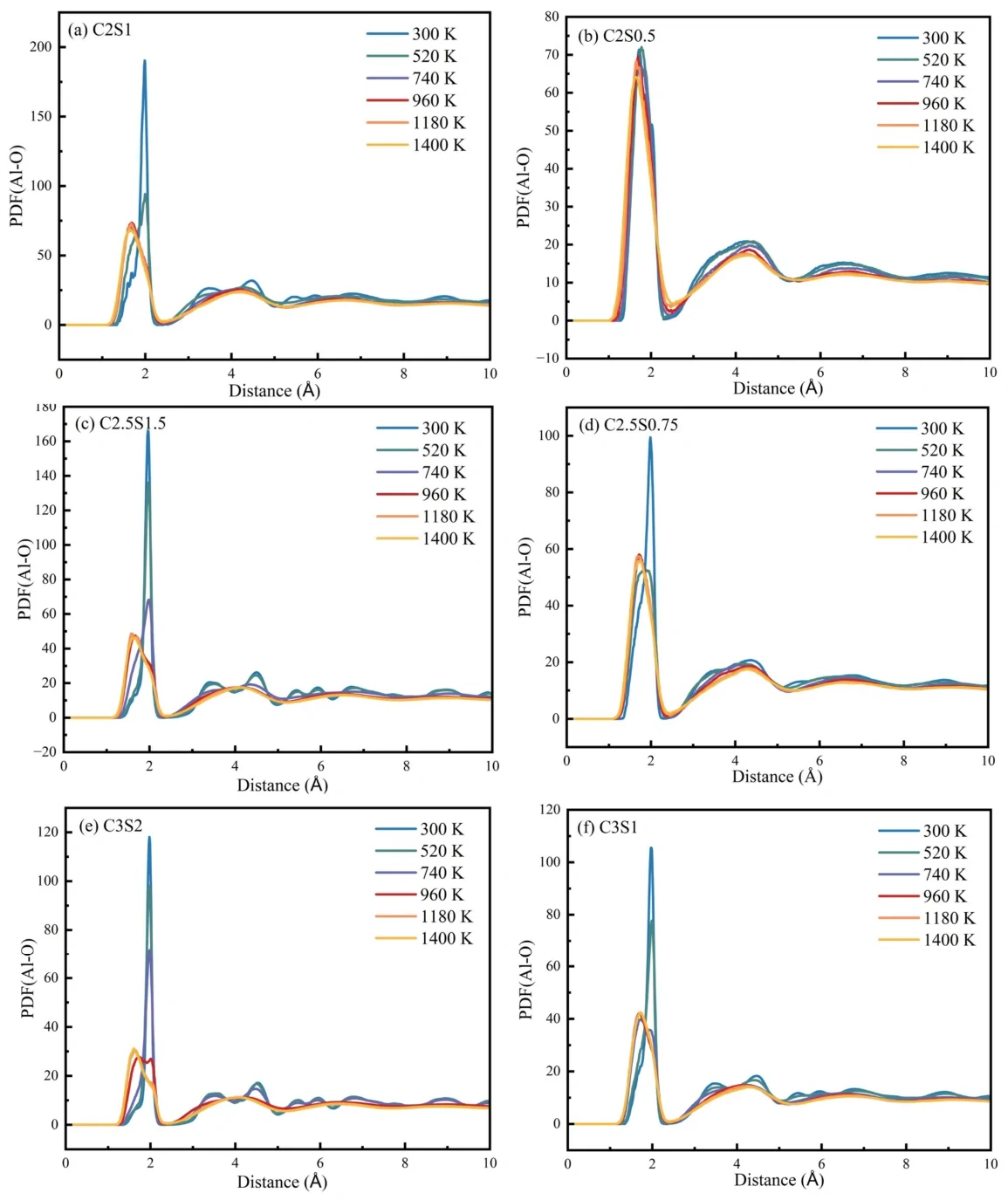
Al–O PDF during the oxidation of (**a**) C2S1, (**b**) C2S0.5, (**c**) C2.5S1.5, (**d**) C2.5S0.75, (**e**) C3S2 and (**f**) C3S1.

To further elucidate the influence of temperature on the oxidation mechanism and the resulting structural evolution, the oxidation behavior of the six aluminum particles during heating was examined using the Al–O pair distribution function, as presented in Figure 10. As the temperature increases, the first PDF peak exhibits a reduction in intensity, a broadening in width, and a shift toward shorter distances, indicating that elevated temperatures promote the amorphization of crystalline aluminum, enhance the short-range order within the amorphous alumina shell, and simultaneously elevate the overall degree of disorder in the system. For the incompletely oxidized thick-shell (Figure 10c,e) and thin-shell (Figure 10d,f) particles, heating to 960 K leads to an increase in the intensity of the first peak for the thin-shell group, whereas the corresponding peak for the thick-shell group continues to decrease. Subsequently, when the consumption of core aluminum atoms reaches a certain threshold or the oxide shell structure attains relative stability, the peak profiles for both groups remain largely unchanged. In the thin-shell group, the shorter diffusion pathways facilitate the outward migration of internal aluminum atoms through the oxide shell, enabling the formation of locally ordered oxide chains or clusters and consequently causing a recovery of the first-peak intensity. In contrast, the thicker oxide shell of the thick-shell group imposes greater diffusional constraints, confining the oxidation reaction to the inner core–shell interface. Under such strong spatial confinement and compressive stress, the newly formed alumina grows inward in a disordered manner, introducing a high density of point defects, dislocations, and localized lattice distortions, which results in a further reduction and broadening of the first PDF peak.

### 3.5. Charge Distribution and Variation in 5–10 nm Aluminum Particles with Different Oxide Shell Thicknesses

#### 3.5.1. Effect of Oxide Shell Thickness on Charge Variation

**Figure 11:**
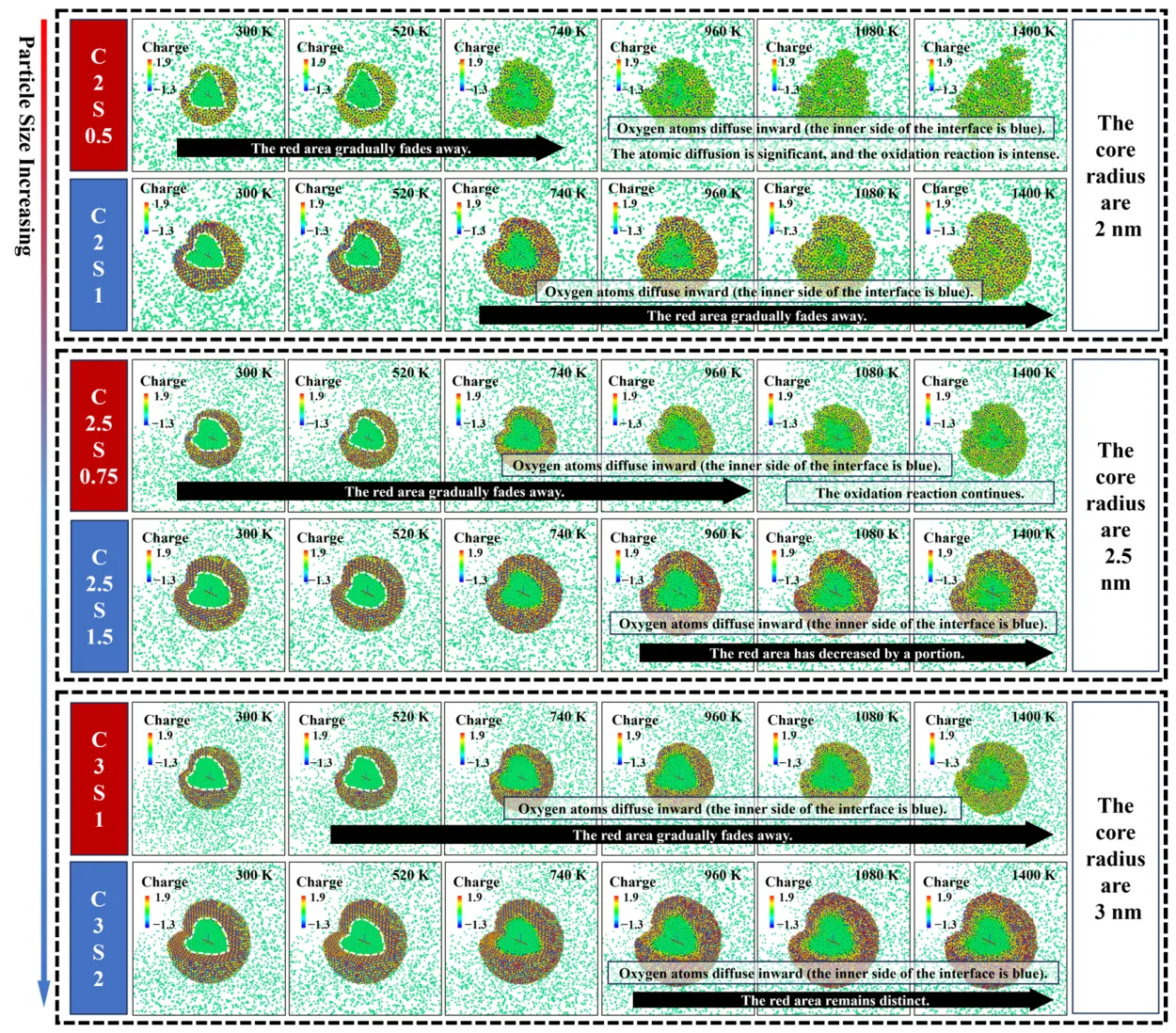
Atomic charge distribution diagram of six aluminum particle models during oxidation process.

The spatial charge distribution directly reflects the electronic environment of the core–shell interface, which significantly influences the diffusion barrier of atoms. In MD simulations, coloring particles according to their charge allows the oxidation status, dynamic charge distribution, and interfacial behavior to be distinguished. Accordingly, charge distribution maps for six aluminum particles (Figure 11) at different temperatures were generated, where red, blue, and green represent positively charged, negatively charged, and neutral particles, respectively. At ambient temperatures (300–520 K), the aluminum core remains neutral (colored green), while the oxide shell and the inner interface display a mixture of positive (Al^3+^/Al^4+^) and negative (O^4−^) ions. The accumulation of negative charge at the internal interface indicates that oxygen inward diffusion precedes aluminum outward diffusion in the initial stage.

For the thin-shell group (C2S0.5, C2.5S0.75, C3S1), the charge gradient across the interface is sharp. As oxidation progresses, the oxide shell gradually loses its charge distinctiveness, transitioning to a uniform charge distribution even at lower temperatures (~960 K). This rapid electron redistribution lowers the potential energy barrier for Al atoms to detach from the core, thus synergistically promoting accelerated atomic diffusion (as verified by the MSD results, with diffusion onset at ~7 ps for thin-shell particles vs. ~15 ps for thick-shell ones, and charge homogenization temperatures of ~960 K vs. ~1400 K, respectively).

In stark contrast, the thick-shell models (C2S1, C2.5S1.5, C3S2) exhibit a persistent charge separation interface. Taking C3S2 as an example, the core remains distinctly neutral and the internal interface maintains a strong negative charge even at 1400 K. The electrostatic stability provided by this thick, uncompromised charge layer imposes an intrinsic energy barrier against metal atom crossover, explaining the delayed onset of diffusion and the retention of a massive neutral Al core in thick-shell configurations.

#### 3.5.2. Effect of Oxide Shell Thickness on Charge Distribution

**Figure 12:**
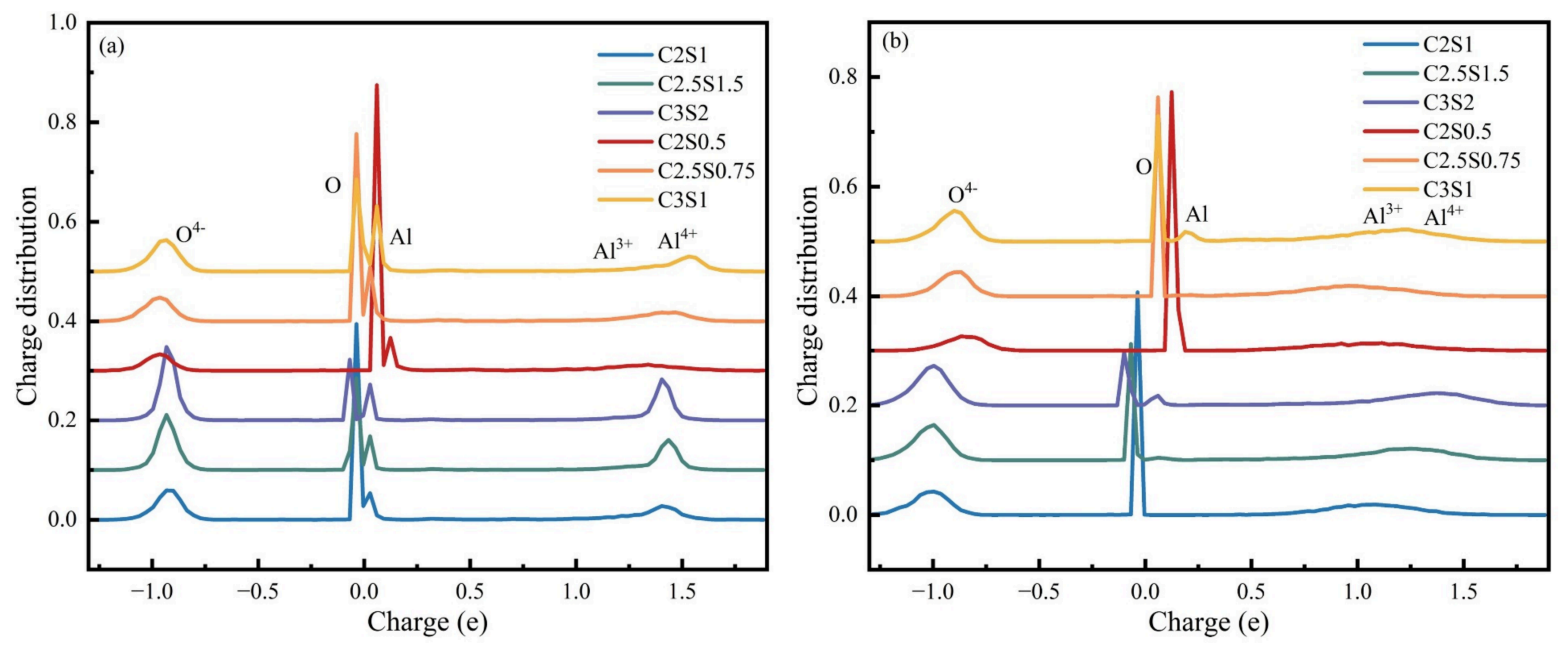
Atomic charge distribution of (**a**) the initial ANPs and (**b**) their final products.

Figure 12 quantitatively compares the atomic charge distributions in the initial state and the final oxide products. Initially, aluminum in the amorphous alumina shell primarily exists as Al^4+^. Upon heating, both Al^3+^ and Al^4+^ ions coexist in the final oxidized state. Specifically, for thin-shell particles (e.g., C2S0.5), all Al is converted to higher valence states after complete oxidation; whereas for thick-shell particles (e.g., C3S2), a significant amount of neutral Al remains at 1400 K, indicating incomplete oxidation. This is highly consistent with the quantitative calculations of active aluminum content (58–84% oxidation fraction). To provide a more quantitative perspective, the relative changes in Al charge states between paired models are compared. For identical core radii, the thin-shell particles exhibit a significantly larger fraction of Al atoms transitioning to higher oxidation states (Al^3+^/Al^4+^) compared to their thick-shell counterparts: for example, the C2S0.5 model shows a ~36.1% increase in shell Al atoms versus ~26.87% for C2S1 (see Figure 5c), accompanied by a more pronounced shift toward higher charges in Figure 12b.

This indicates that the thinner the initial oxide shell, the easier it is for the aluminum core to undergo interfacial electron loss. The enhanced charge transfer observed in thin-shell particles correlates directly with their earlier MSD onset and faster active aluminum consumption. Therefore, the initial oxide shell thickness dictates the charge transfer efficiency, which serves as an underlying electronic-level driver for the macroscopic oxidation rate.

The observed correlation between charge redistribution and atomic diffusion at the core–shell interface can be fundamentally rationalized by Sharma et al. [29] systematical investigation. Specifically, their findings revealed that efficient interfacial charge transfer reduces the potential barrier and facilitates ion diffusion, whereas a persistent charge separation layer imposes a kinetic barrier that suppresses interfacial reactions. This mechanistic interpretation directly supports our MD simulation results: the thin-shell models (C2S0.5, etc.) exhibit rapid spatial charge homogenization and enhanced electron loss from Al atoms (transition to higher oxidation states), which effectively lowers the diffusion barrier and accelerates the outward Al migration. In contrast, the thick-shell models sustain a distinct charge separation layer, analogous to the electrostatically stabilized interface reported by Sharma et al., thereby impeding oxygen inward penetration and limiting the overall oxidation fraction.

## 4. Conclusions

This study utilizes ReaxFF molecular dynamics simulations to reveal the atomistic oxidation mechanisms of core–shell ANPs during heating condition. By systematically coupling morphological evolution with atomic stress and charge redistribution analysis, the following mechanistic insights are reached:

(1) Particle size and oxide shell thickness synergistically govern oxidation completeness. Smaller core sizes and thinner shells accelerate atomic diffusion and increase the oxidation fraction (up to 100% for the C2S0.5 model at 1400 K). The MSD analysis confirms that atomic migration in smaller particles initiates earlier (~7 ps) and proceeds more vigorously, driven by the enhanced thermal vibration at the surface.

(2) The variation in internal compressive-to-tensile stress determines the oxidation pathway. Thin oxide shells are prone to stress-induced rupture. The outward diffusion of molten Al creates internal pressure, which, combined with thermal expansion, converts the initial compressive stress into tensile stress. When this tensile strength is exceeded (~1060 K for C2S0.5), shell rupture occurs, leading to the rapid formation of detached alumina clusters. Conversely, thick shells maintain compressive confinement, which physically suppresses core diffusion and prevents structural rupture, though it limits the overall oxidation degree to 58–84%.

(3) The interfacial charge transfer acts as the electronic driving force for oxidation. Thin-shell particles exhibit rapid charge homogenization and enhanced electron loss from Al^3+^/Al^4+^ state transitions, lowering the electrostatic barrier for atomic transport. In contrast, thick-shell configurations maintain a distinct charge separation layer, impeding oxygen penetration and Al outward migration.

(4) While specific numerical values of oxidation may change under different oxygen concentrations, the revealed physical mechanism (stress-driven shell rupture and charge redistribution-driven interfacial diffusion) should remain universal.

(5) Macroscopic oxidation behavior is dominated by the coupled effects of temperature-driven diffusion, stress evolution, and charge redistribution. Our findings provide an atomic-scale mechanistic framework for understanding the heating hazards of aluminum powders, offering crucial theoretical guidance for designing safer core–shell structures with tailored oxide thicknesses for industrial applications.

## Figures and Tables

**Table 1:** Aluminum particle model settings.

Group Number	Name	Aluminum Core Radius/nm	Aluminum Oxide Shell Thickness/nm	Particle Size/nm
1-thick	C2S1	2.0	1.0	6.0
	C2.5S1.5	2.5	1.5	8.0
	C3S2	3.0	2.0	10.0
2-thin	C2S0.5	2.0	0.5	5.0
	C2.5S0.75	2.5	0.75	6.5
	C3S1	3.0	1.0	8.0

## Data Availability

The participants of this study did not give written consent for their data to be shared publicly, so due to the sensitive nature of the research supporting data is not available.

## Abbreviations

The following abbreviations are used in this manuscript:

ANPs	Aluminum nanoparticles
MSD	Mean squared displacement
MD	Molecular dynamics simulations
PDF	Pair distribution function
FCC	Face-centered cubic
